# Intraoperative Contrast‐Enhanced Ultrasonography (Io‐CEUS) to Detect Missed Lung Nodules During Robot‐Assisted Thoracoscopic Surgery

**DOI:** 10.1111/1759-7714.70377

**Published:** 2026-08-26

**Authors:** Alfonso Fiorelli, Antonio Marella, Marco Aiello, Valentina Brancato, Marco Salvatore, Daniela Comunale, Francesco Ferrigno, Gaetana Messina

**Affiliations:** ^1^ Thoracic Surgery Unit University of Campania “Luigi Vanvitelli” Naples Italy; ^2^ IRCCS Synlab SDN Naples Italy; ^3^ Oncology Unit Salerno Hospital Salerno Italy; ^4^ ASL SA Salerno Italy

**Keywords:** CEUS, intra‐operative ultrasound, lung cancer, robotic surgery

## Abstract

Herein, we reported the use of Intraoperative‐Contrast Enhanced Ultrasonography (Io‐CEUS) during Robotic Assisted Thoracic Surgery (RATS) for identification of malignant lung nodule that was missed during previous operation. Pre‐operative CT scan with 3D reconstruction defined the prompt position of the target lesion within S8 segment of right lower lobe and the anatomic landmarks as the distance from interlobar fissure and diaphragm. The lesion was then successfully detected by Io‐CEUS. The different arterial enhancement observed during wash‐in (hyperenhancement) and wash‐out (hypo‐enhancement) phases helped to differentiate the nodule from lung parenchyma. The lesion was resected by mechanical stapler and frozen section analysis diagnosed metastasis from colon carcinoma. Post‐operative course was unremarkable; patient was discharged on post‐operative day 3.

## Graphical abstract

During robotic thoracic surgery, Intraoperative Ultrasound is the surgeon's finger to detect small nodule.

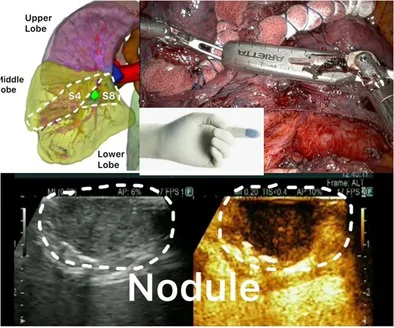

## 1 Introduction

Detection of solitary pulmonary nodules (SPNs) during minimally invasive surgery (MIS) may be challenging when the lesions are small and deeply located [1, 2]. Herein, we reported the use of Intraoperative‐Contrast Enhanced Ultrasonography (Io‐CEUS) during Robotic Assisted Thoracic Surgery (RATS) for identification of malignant lung nodule that was missed during previous operation performed by thoracotomy.

## 2 Case Description

**FIGURE 1:**
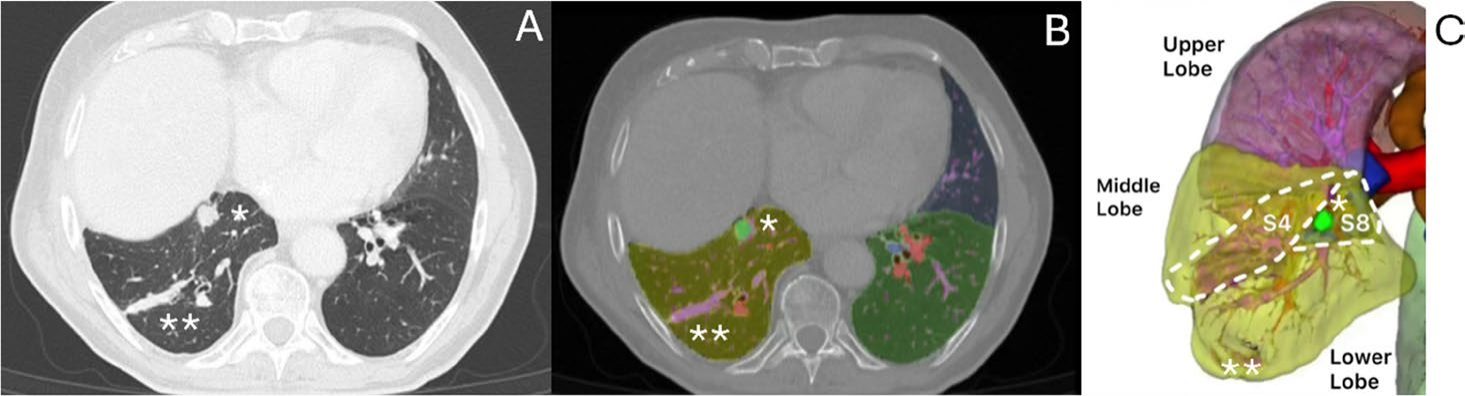
Chest computed tomography scan (A) and 3D segmentation (B) showed tumor (*) and previous resections (**). 3D model (C) defined that prompt position of the tumor (*) and previous resection (**).

A 77‐year‐old man with a past medical history of colon carcinoma, diabetes and hypertension underwent surgical resection of lung nodule localized in RLL, highly suspicious for malignancy, at another hospital. The nodule was missed during thoracoscopy and a conversion to thoracotomy was performed. However, a wrong lesion was resected and postoperative CT scan 1 month later confirmed the presence of the same nodule (Figure 1A). The lesion presented pathological uptake on the FDG‐PET scan. No other pathological lesions were found. The patient was referred to our hospital where he was scheduled for re‐operation after MDT discussion. The procedure was preoperatively planned by 3D reconstruction model. It showed that the lesion was contained within anterior segment (S8) of RLL (Figure 1B), close to the anterior part of the oblique fissure, and in proximity to the diaphragm surface (Figure 1C).

RATS resection was planned. Before docking the robot, a 2 cm incision was performed at the level of 5th intercostal space, through that ultrasonic energy device (Harmonic, Ethicon Inc., Cincinnati, OH) was inserted into the chest cavity. The adhesions between the lung and the chest wall were dissected to create access to the pleural cavity for insertion of the robotic' trocars. Then, four incisions were performed at the level of the 8th intercostal space, and a standard 4‐arm approach was used for the procedure. Firstly, the lung was detached from the chest wall using a combination of blunt, sharp and electrocautery dissection and anatomic landmarks as azygous vein and vena cava were identified. Then, the lung was separated from the pericardium and the mediastinum, and anatomic landmarks such as phrenic nerve and pulmonary veins were identified. Finally, the lung was separated from the diaphragm and fully mobilized.

**FIGURE 2:**
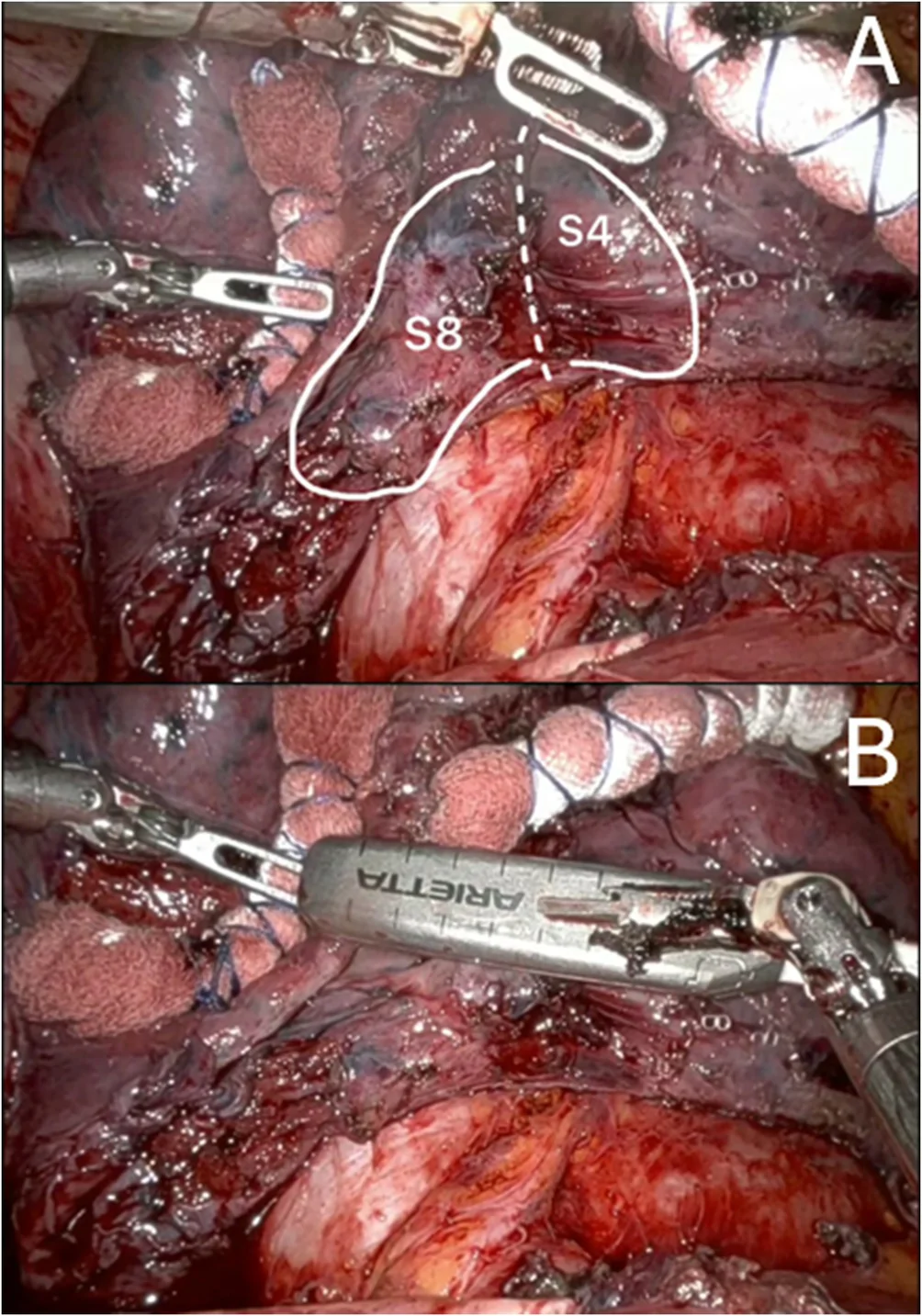
The target segment was intraoperatively identified (A) and scanned by ultrasound (B) to detect tumor.

**FIGURE 3:**
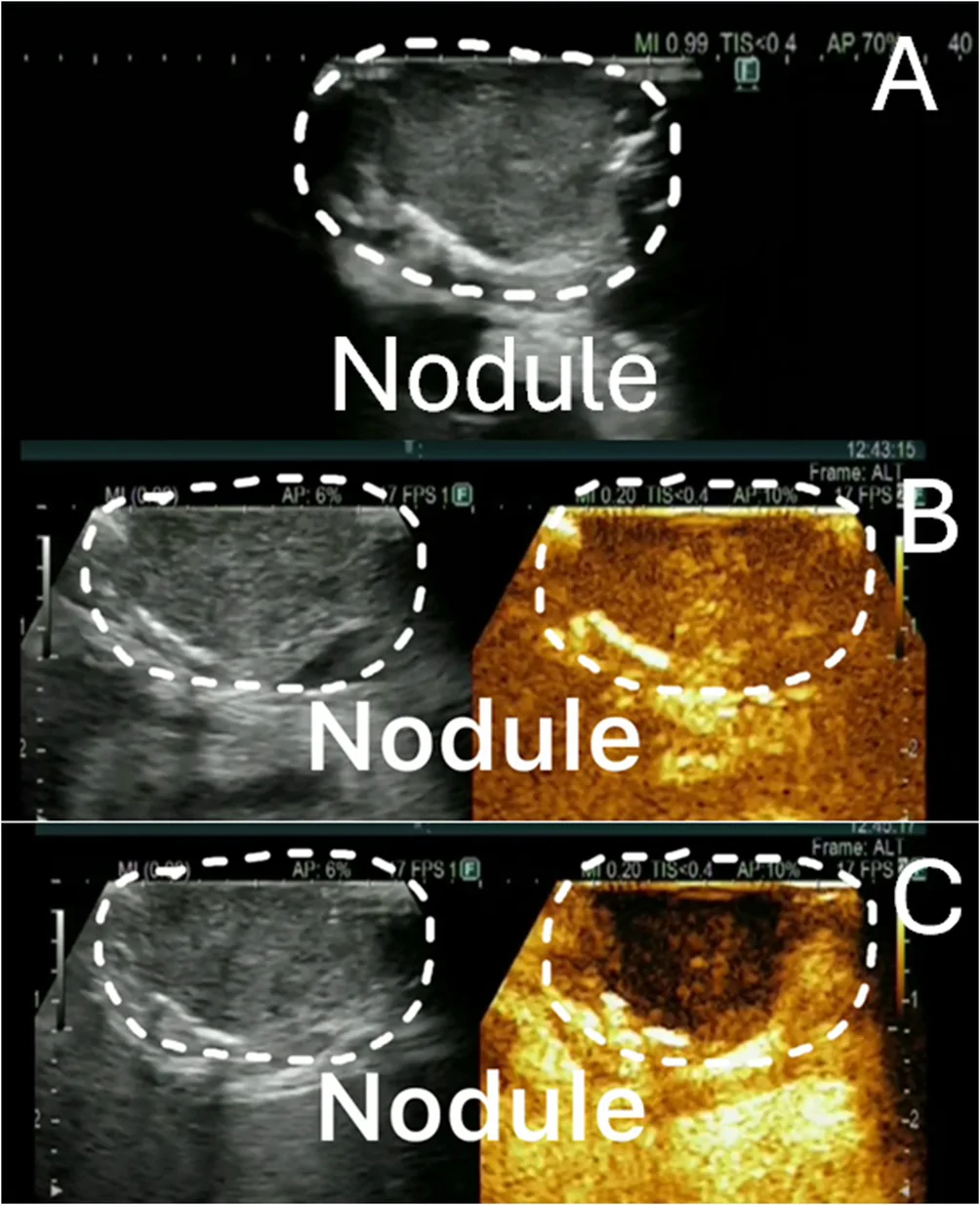
The nodule was identified as a hypoechoic lesion by ultrasound (A). After administration of contrast‐enhanced, it presented hyper‐enhancement during wash‐in phase (B) and hypo‐enhancement during wash‐out phase (C).

Then, ultrasound probe (L51K Robotic Probe, 15 MHz, 13 mm Linear Probe) dedicated for Da Vinci Systems was inserted into the chest through the assistant port and fixed with a bipolar clamp to scan the anterior segment of lower lobe (S8) having as anatomic landmarks the anterior part of oblique fissure and the inferior pulmonary vein (Figure 2A,B). The nodule was identified as a hypoechoic lesion (Figure 3A). Then, 2.4–5 mL contrast‐enhanced (SonoVue, Bracco, Milan, Italy) followed by 10–20 mL of NaCl was administered and the lesion perfusion was scanned continuously for at least 3 min. The US findings of the lesion changed based on the blood flow. The lesion presented marked uptake (hyper‐enhancement) due to the high arterial blood flow during wash‐in phase (Figure 3B) and then absence of uptake (hypo‐enhancement) compared to normal lung parenchyma during wash‐out phase (Figure 3C). The surface of the nodule was catheterized by bipolar forceps. The anterior part of the oblique fissure was developed by mechanical stapler, and the nodule was then resected. Frozen section analysis diagnosed metastasis from colon carcinoma with negative margins. Thus, lobectomy was not performed. Chest drainage was left in pleural cavity. The operation was summarized in Video S1.

Postoperative course was unremarkable. Chest drainage was removed on postoperative day two and patient discharged the day after. At time of this report, the patient was well and followed by oncologist.

Written informed consent was obtained from patient for this publication.

## 3 Discussion

The use of low‐dose CT scan in the follow‐up of oncological patients has increased the diagnosis rate of SPNs [1]. Surgical resection by MIS remained the strategy of choice when other less invasive strategies such as CT‐guided biopsy or EBUS‐bronchoscopy failed to obtain a definitive diagnosis. However, detection of SPN may be challenging during MIS. Missing a lung nodule during the initial wedge resection could occur in 1%–5% of cases [1, 2] and needed additional incisions or conversion from MIS to thoracotomy to facilitate detection by manual palpation, as in the present. Despite all, the target nodule was misidentified, and a wrong lesion was resected. The nodule was sub‐centimetric, deep and located in a convex part of the lung (S8 segment near to the diaphragm surface) difficult to palpate. Furthermore, no pre‐operative and/or intraoperative strategies to detect it were performed. All these factors likely facilitated the misidentification of the target lesion.

The wrong resection of neoplastic lung nodule is a clinical event that happens in real‐world clinical practice but under‐reported in literature. The patient should be scheduled for early re‐operation to prevent growth and/or dissemination of neoplastic lesion, as in the present. The surgical approach and the intraoperative detection of the target nodule were the main issues in this case. MIS was particularly indicated in this frail patient, but the presence of dense adhesions due to previous thoracotomy could be a relative or absolute contraindication for this approach. Furthermore, previous lung resections distorted the lung parenchyma limiting the identification of anatomic landmarks that could help the detection of target lesion.

RATS was considered the more appropriate approach. From a clinical point of view, it preserved all advantages of MIS compared to thoracotomy, while, from a technical point the wristed robotic instruments and the 3D operative view made easier the lysis of adhesions and scar tissue compared to thoracoscopy. Similarly, Ricciardi et al. [3] showed that RATS was a safe and feasible approach to perform lung resection and thymectomy in selected patients undergoing previous thoracotomy.

The main limitation of RATS was the lack of tactile feedback for identifying the nodule. The pre‐operative marking of the lesion with hook‐wire using CT scan was at high risk of complications as the nodule was localized close to mediastinum and diaphragm [4]. Furthermore, electromagnetic navigation bronchoscopy [5] to place coils or to inject dyes like indocyanine green (ICG) into the lesion was not available in our hospital. Conversely, Io‐US did not require invasive procedures as the probe was placed directly on lung parenchyma, providing real‐time imaging of the lesion. Furthermore, it was widely accessible in the operating room as the present intraoperative US‐probe was cleared for general and urologic robotic surgery procedures [6] while its use in thoracic surgery was still limited [7, 8]. In line with previous experiences [1, 2], a 3D reconstruction model preoperatively identified the position of the lesion and defined the anatomical landmarks that then facilitated the intra‐operative detection by CEUS. Previous authors [7, 8] used Io‐US for lung nodule detection during RATS, but no paper, before the present, reported the use of Io‐CEUS for this scope. Compared to Io‐US [7, 8], Io‐CEUS provided continuous monitoring of blood flow lesion in real time [9, 10]. The different arterial enhancement observed during wash‐in (hyperenhancement) and wash‐out (hypo‐enhancement) phases helped to differentiate the nodule from lung parenchyma. Similarly, Wang et al. [9] and Di Vece et al. [11] found that CEUS assessment before trans‐bronchial biopsy increased the detectable rate of lesions hidden in atelectasis lungs compared to traditional US. Compared to the above reported strategies for detection SPN, Io‐CEUS presented several limits. (i) Emphysema and inflated lung could limit the US ability to detect lung nodules as the air acted as a barrier to ultrasound waves. Pressure the lung parenchyma with a probe and continued suction of airways by anesthetists during the procedure helped to deflate the lung. Furthermore, the use of saline solution improved the adhesion of US probe with lung parenchyma, enhancing the transduction of ultrasound waves and limiting the artifacts. (ii) US was operator‐dependent, and the results depended strongly on the skill of surgeons. Thus, radiologists or thoracic surgeons with US skills should supervise the first procedures performed by surgeons with limited US experience. (iii) Scanning the entire lobe by US to detect lung lesions was a time‐consuming procedure that could overcome by the preoperative identification of anatomic landmarks by preoperative 3D CT scan reconstruction.

Finally, our case underscored a clinical situation under‐reported in literature as the resection of wrong nodule. In case of reoperation, RATS was a safe and feasible procedure that facilitated the nodule resection also in presence of tenacious adhesions due to previous thoracotomy and preserved the advantage of MIS. Furthermore, Io‐CEUS allowed in real time the identification of small and deep lesions also localized in “obscure” parts of the lung difficult to palpate and distorted by previous lung resections.

## Author Contributions

**Marco Aiello:** data curation. **Valentina Brancato:** data curation. **Daniela Comunale:** data curation. **Marco Salvatore:** supervision. **Francesco Ferrigno:** data curation. **Alfonso Fiorelli:** writing – original draft. **Gaetana Messina:** writing – review and editing. **Antonio Marella:** data curation.

## Funding

The authors have nothing to report.

## Consent

An informed written consent was obtained by the patients for the publication of the study.

## Conflicts of Interest

The authors declare no conflicts of interest.

## Supporting information


**Video S1:** The video summarized the main steps of operation as the resection of adhesions and the mobilization of the lung; the handling and manipulation of the ultrasound probe; the US detection of the lesion before and administration of contrast enhanced; and the tumor resection by dedicated robotic stapler.

## Data Availability

The data that support the findings of this study are available on request from the corresponding author. The data are not publicly available due to privacy or ethical restrictions.

## References

[1] J. Whooley , R. Weedle , D. Breen , and A. Soo , “Lung Cancer Resection in the Absence of Pre‐Operative Histology: The Accuracy of Multidisciplinary Team Consensus,” European Journal of Surgical Oncology 49, no. 9 (2023): 106907. 37080864 10.1016/j.ejso.2023.04.006

[2] G. Natale , B. Leonardi , G. Messina , et al., “Three‐Dimensional Lung Reconstructions for the Localization of Lung Nodules To Be Resected During Surgery,” Thorac Cancer 14, no. 34 (2023): 3389–3396. 37860943 10.1111/1759-7714.15131 PMC10693940

[3] S. Ricciardi , C. C. Zirafa , F. Davini , and F. Melfi , “How to Get the Best From Robotic Thoracic Surgery,” Journal of Thoracic Disease 10 (2018): S947–S950. 29744221 10.21037/jtd.2018.03.157 PMC5934117

[4] Z. D. Zhang , H. L. Wang , X. Y. Liu , F. F. Xia , and Y. F. Fu , “Methylene Blue Versus Coil‐Based Computed Tomography‐Guided Localization of Lung Nodules,” Thoracic and Cardiovascular Surgeon 68, no. 6 (2020): 540–544. 32311745 10.1055/s-0040-1708836

[5] L. Zhang , M. Li , Z. Li , et al., “Three‐Dimensional Printing of Navigational Template in Localization of Pulmonary Nodule: A Pilot Study,” Journal of Thoracic and Cardiovascular Surgery 154, no. 6 (2017): 2113–2119.e7. 29017792 10.1016/j.jtcvs.2017.08.065

[6] https://www.accessdata.fda.gov/cdrh_docs/pdf22/K223830.pdf.

[7] Z. Zhou , Z. Wang , Z. Zheng , et al., “An “Alternative Finger” in Robotic‐Assisted Thoracic Surgery: Intraoperative Ultrasound Localization of Pulmonary Nodules,” Medical Ultrasonography 19, no. 4 (2017): 374–379. 29197913 10.11152/mu-1053

[8] A. Fiorelli , S. Forte , V. Di Filippo , et al., “Lung Nodule Detection by Intraoperative Ultrasound During Robotic Surgery,” JTCVS Techniques 30 (2025): 148–150. 40242118 10.1016/j.xjtc.2025.01.028 PMC11998323

[9] S. Wang , W. Yang , H. Zhang , Q. Xu , and K. Yan , “The Role of Contrast‐Enhanced Ultrasound in Selection Indication and Improveing Diagnosis for Transthoracic Biopsy in Peripheral Pulmonary and Mediastinal Lesions,” BioMed Research International 2015 (2015): 231782. 26090391 10.1155/2015/231782 PMC4450237

[10] S. Jiménez‐Serrano , A. Páez‐Carpio , B. Doménech‐Ximenos , et al., “Conventional and Contrast‐Enhanced US of the Lung: From Performance to Diagnosis,” Radiographics 44, no. 7 (2024): e230171. 38935548 10.1148/rg.230171

[11] F. di Vece , P. Tombesi , F. Ermili , and S. Sartori , “Contrastenhanced Ultrasound (CEUS) and CEUS‐Guided Biopsy in the Diagnosis of Lung Abscess in a Patient With Achalasia: Case Report,” Interventional Medicine and Applied Science 5, no. 1 (2013): 31–33. 24265886 10.1556/IMAS.5.2013.1.6 PMC3831797

